# Dehydrocostuslactone Suppresses Angiogenesis *In Vitro* and *In Vivo* through Inhibition of Akt/GSK-3β and mTOR Signaling Pathways

**DOI:** 10.1371/journal.pone.0031195

**Published:** 2012-02-16

**Authors:** Chih-Ya Wang, An-Chi Tsai, Chieh-Yu Peng, Ya-Ling Chang, Kuo-Hsiung Lee, Che-Ming Teng, Shiow-Lin Pan

**Affiliations:** 1 Pharmacological Institute, College of Medicine, National Taiwan University, Taipei, Taiwan; 2 Institute of Biotechnology and Pharmaceutical Research, National Health Research Institutes, Zhunan Town, Taiwan; 3 Natural Medicinal Products Research Center, China Medical University Hospital, Taichung, Taiwan; 4 Graduate Institute of Integrated Medicine, College of Chinese Medicine, China Medical University, Taichung, Taiwan; 5 Natural Products Research Laboratories, Eshelman School of Pharmacy, University of North Carolina, Chapel Hill, North Carolina, United States of America; 6 Chinese Medicine Research and Development Center, China Medical University and Hospital, Taichung, Taiwan; Wayne State University School of Medicine, United States of America

## Abstract

The traditional Chinese medicine component dehydrocostuslactone (DHC) isolated from *Saussurea costus* (Falc.) Lipschitz, has been shown to have anti-cancer activity. Angiogenesis is an essential process in the growth and progression of cancer. In this study, we demonstrated, for the first time, the anti-angiogenic mechanism of action of DHC to be via the induction of cell cycle progression at the G0/G1 phase due to abrogation of the Akt/glycogen synthase kinase-3β (GSK-3β)/cyclin D1 and mTOR signaling pathway. First, we demonstrated that DHC has an anti-angiogenic effect in the matrigel-plug nude mice model and an inhibitory effect on human umbilical vein endothelial cell (HUVEC) proliferation and capillary-like tube formation *in vitro*. DHC caused G0/G1 cell cycle arrest, which was associated with the down-regulation of cyclin D1 expression, leading to the suppression of retinoblastoma protein phosphorylation and subsequent inhibition of cyclin A and cdk2 expression. With respect to the molecular mechanisms underlying the DHC-induced cyclin D1 down-regulation, this study demonstrated that DHC significantly inhibits Akt expression, resulting in the suppression of GSK-3β phosphorylation and mTOR expression. These effects are capable of regulating cyclin D1 degradation, but they were significantly reversed by constitutively active myristoylated (myr)-Akt. Furthermore, the abrogation of tube formation induced by DHC was also reversed by overexpression of Akt. And the co-treatment with LiCl and DHC significantly reversed the growth inhibition induced by DHC. Taken together, our study has identified Akt/GSK-3β and mTOR as important targets of DHC and has thus highlighted its potential application in angiogenesis-related diseases, such as cancer.

## Introduction

Angiogenesis, which is the process of formation of new blood vessels from pre-existing ones, takes place throughout physiological development, tissue repair, and reproduction [1]. In pathological conditions, angiogenesis is also essential for tumor growth and progression to ensure that more oxygen and nutrients are delivered from the host's vascular system. The induction of tumor vasculatures, termed the angiogenic switch, is initiated by various growth factors (vascular endothelial growth factor (VEGF) and basic fibroblast growth factor (bFGF) are the most commonly ubiquitous) released from tumor cells, and is tightly regulated by the balance of angiogenic activators and inhibitors [2]. The angiogenic factors stimulate the degradation of extracellular matrix components of the parent vessels, which allow endothelial cells to migrate, proliferate, and form tube-like structures. After forming new capillary sprouts, the blood vessels mature. Thus, inhibition of the steps of angiogenesis by blocking angiogenesis-related proteins could be a strategy to arrest tumor growth [3].

Akt is a serine/threonine protein kinase activated by growth factors, which transmits survival signals to downstream effectors. In endothelial cells, the major growth factor-induced angiogenic responses are mediated predominantly by Akt signaling [4]. Activation of the Akt signaling pathway is critical for cell proliferation and growth of endothelial cells. After receptor stimulation, Akt targets a number of established substrates for phosphorylation. One of the major effectors downstream of Akt is glycogen synthase kinase (GSK)-3 [5], [6]. Upon the activation of Akt, Akt inactivates GSK-3β by initiating phosphorylation by at its serine 9 residue. GSK-3β is thought to regulate cell cycle progression by phosphorylating cyclin D1 at the threonine 286 residue. This later promotes nuclear export and targets the protein for ubiquitylation and subsequent degradation by proteasomes [7], [8]. Therefore, growth factors promote cyclin D1 stabilization and up-regulation through the Akt/GSK-3β pathway [9]. Cyclin D1 is an essential cell cycle regulatory molecule, which allows G1/S phase entry and cell division by binding to cyclin-dependent kinase (Cdk) 4. Cyclin D1–Cdk4 phosphorylates the retinoblastoma (Rb) protein, upon which E2F is released to transactivate the genes required for the G1- to S-phase progression [10], [11]. Overexpression of cyclin D1 is frequently associated with tumorigenesis, angiogenesis, and tumor growth [12]. Recently, accumulating studies have demonstrated that cyclin D1 inhibition leads to endothelial cell growth arrest, such as cyclin D1 antisense, which significantly suppressed VEGF-induced *in vitro* tube formation and tumor-associated neo-vascularization in the matrigel plug assay and tumor xenograft mouse models [13].

Dehydrocostuslactone (DHC), a sesquiterpene lactone derivative, is an active component isolated from *Saussurea costus* (Falc.) Lipschitz, which shows potential anti-inflammatory [14], hepatoprotective [15], immunomodulatory [16], [17], and cholagogic effects, and is used in the treatment of ulcers [18]. Recently, DHC has been reported to potentiate anti-proliferative effects in ovarian cancer cells [19] and induce apoptosis in various tumors. This occurs as a result of DHC preventing tumor necrosis factor (TNF)-α-induced degradation and phosphorylation of IκBα in human promyelocytic leukemia cells (HL-60) [20], induction of endoplasmic reticulum stress in hepatoma and lung cancer [21], [22], and inhibition of signal transducers and activators of transcription 3 (STAT3) in breast cancer [23]. Although DHC has already been shown to be promising for tumor suppression *in vitro* and *in vivo*, whether it can modulate tumor angiogenesis has not been validated yet. This study elucidated the molecular mechanism involved in the anti-proliferative response induced by DHC in endothelial cells. We demonstrated that DHC inhibits angiogenesis through the Akt/GSK-3β and mTOR pathways. Furthermore, the *in vivo* matrigel plug assay indicated that DHC is a potential anti-angiogenic agent.

## Results

### Effect of DHC on angiogenesis *in vivo*

**Figure 1:**
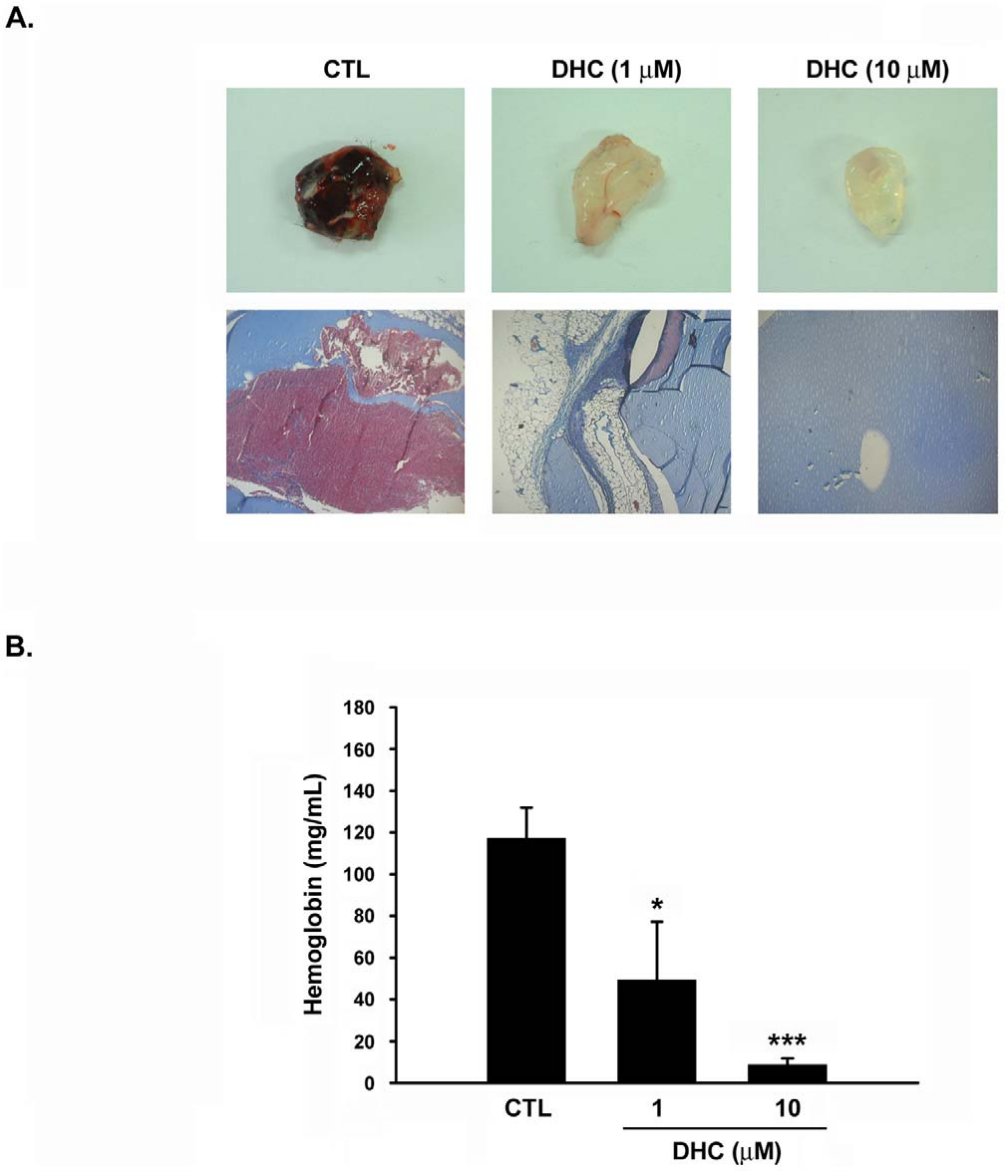
DHC inhibits angiogenesis in a mouse matrigel model. C57BL/6 mice were injected subcutaneously with matrigel mixed with or without DHC (1 µM and 10 µM). A, upper panel, plugs were excised from the mice after a week and photographed. Bottom panel, histological analysis was performed using Masson-Trichrome staining from the excision of matrigel plug. B, Quantification of the hemoglobin content of matrigel plugs by spectrophotometer measured at 540 nm. Data represent the mean ± SEM from five independent experiments. * *P*<0.05 and *** *P*<0.001 versus control.

To determine the role of DHC in the regulation of angiogenesis *in vivo*, we analyzed matrigel plug formation following subcutaneous implantation in mice. Matrigel mixed with angiogenic growth factors induced blood vessel growth and showed blood cell-filled angiogenic vasculature. In contrast, DHC significantly inhibited neo-vascularization in a concentration-dependent manner (Figure 1A). Following Masson's trichrome staining, the vasculature within the plugs identified more vessel-like structures within the control group than in the DHC-treated groups. This study then quantified the vascular formation in the matrigel by analyzing the hemoglobin content. The plugs mixed with a variant of growth factors contained 117.3 mg/mL of hemoglobin, identical to the formation of functional vasculature in the matrigel (Figure 1B). Co-treatment with DHC reduced the hemoglobin content in the plugs in a concentration-dependent manner, demonstrating that DHC has potential inhibitory activity in growth factors-induced angiogenesis *in vivo*.

### Effect of DHC on angiogenesis *in vitro*

**Figure 2:**
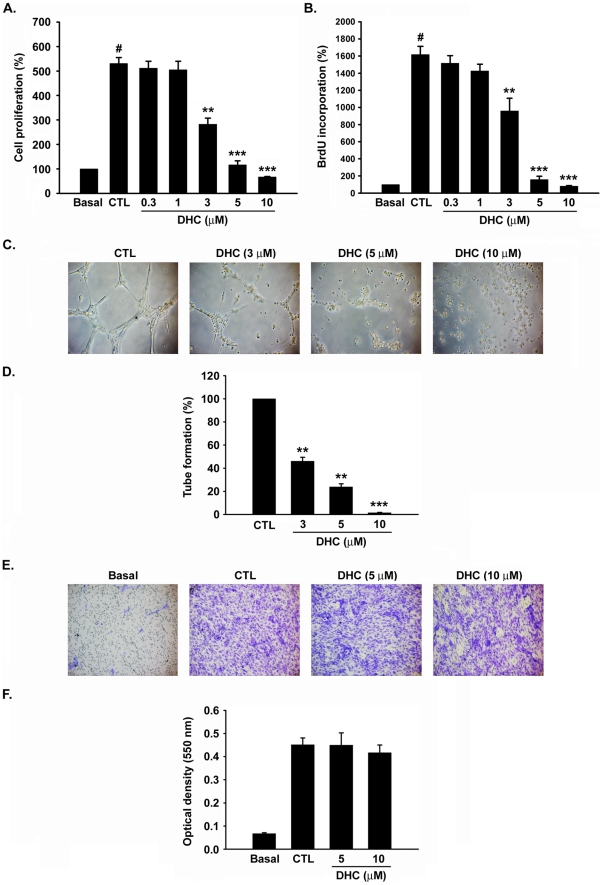
Impairment of *in vitro* angiogenesis by DHC. A, HUVECs were treated with or without DHC (0.3–10 µM) in EGM-2 medium. After 72 h of incubation, cells were stained with crystal violet and determined the inhibition of cell proliferation by the absorbance at 550 nm. B, DNA synthesis was assessed by BrdU incorporation assay. C, representative photographs of capillary-like structures formation of CTL and DHC-treated HUVECs on matrigel under microscope (magnification is X100). D, Quantification of the total tube length of capillary-like structures by image analysis software. Data represent the mean ± SEM from three independent experiments. ** *P*<0.01 and *** *P*<0.001 versus control. E, effect of DHC on cell migration using a transwell assay. F, the graph shows quantitative analysis of the migrated cell numbers in tranwell assay. Data represent the mean ± SEM from three independent experiments. ## *P*<0.01 versus basal.

In angiogenic processes, endothelial cells must undergo ECM degradation, migration, proliferation, and tube formation to form new blood vessels [2]. To investigate whether DHC inhibits angiogenesis through these steps *in vitro*, the well-established functional assays were executed. In the crystal violet assay and the 5-bromo-2-deoxyuridine (BrdU) incorporation labeling assay, DHC (0.3–10 µM) inhibited endothelial growth medium (EGM)-2–induced cell proliferation and DNA synthesis in a concentration-dependent manner (GI_50_ = 2.4 and 3.0 µM, respectively) (Figure 2A and 2B). Next, the effects of DHC on the morphological differentiation and migration of HUVECs were examined. DHC was found to inhibit the formation of capillary-like structures induced by EGM-2 in a concentration-dependent manner. Quantification of the tubes, counted in 5 random fields under a microscope, showed a 54.0% (*P*<0.001), 76.2% (*P*<0.001), and 98.6% (*P*<0.001) inhibition following treatment with 3, 5, and 10 µM DHC, respectively (Figure 2C and 2D). However, DHC had no significant effect on cell migration in a transwell assay (Figure 2E and 2F). Overall, these results indicated that DHC has potent anti-angiogenic activity *in vitro* and *in vivo*.

### DHC causes G0/G1 phase cell cycle arrest in endothelial cells

**Figure 3:**
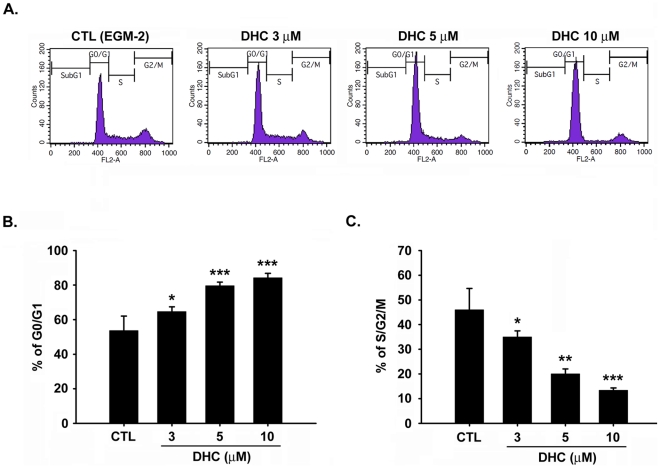
Effect of DHC on cell cycle. A, HUVECs were starved for 24 h, and then were treated with or without DHC (3–10 µM) for 18 hr. After cells were labeled with propidium iodide, DNA content was analyzed by flow cytometry. B and C, cell population in G0/G1 and S/G2/M phase were quantified. Data represent the mean ± SEM from four independent experiments. * *P*<0.05, ** *P*<0.01 and *** *P*<0.001 versus control.

**Figure 4:**
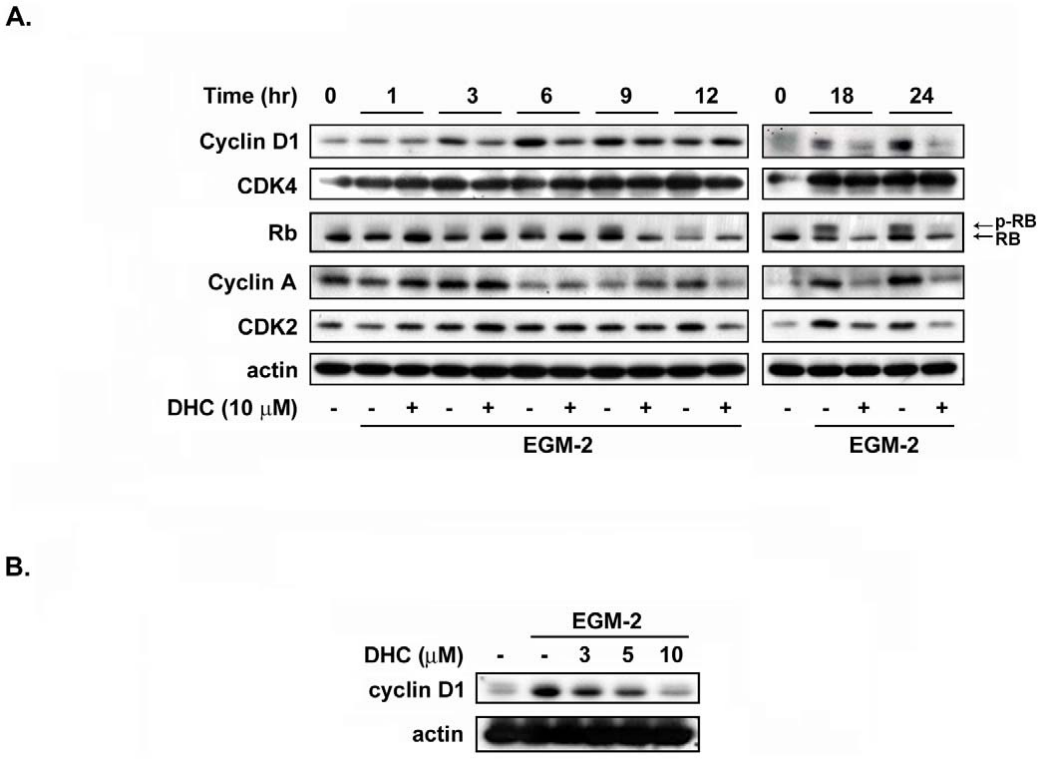
Effect of DHC on expression levels of cyclins and CDKs. A, after starvation for 24 hr, HUVECs were pretreated with or without DHC for 60 min and then incubated with EGM-2 at indicated time. Cells were harvested, analyzed by western blot and probe with antibodies. B, cyclin D1 expression was inhibited by DHC in a concentration dependent manner. Actin expression served as a loading control. Data represent from three independent experiments.

To examine the impaired cell proliferation induced by DHC, this study utilized flow cytometry to determine whether the DHC-mediated growth inhibition was due to cell cycle arrest. Treatment of the cells with DHC (3–10 µM) for 18 h increased the percentage of HUVECs in the G0/G1 phase and decreased the population of cells in the S/G2/M phase (Figure 3A, 3B, and 3C). We also examined the apoptotic effect of DHC under serum starvation. However, there is no significant difference between control cells and DHC-treated cells under serum starved condition (Figure S4). The apoptotic effect in DHC-treated group might be induced by the serum and growth factors withdrawal. Next, the effect of DHC on the expression of cell cycle-regulated proteins involved in the G0/G1 phase was examined. As shown in Figure 4A and 4B, DHC significantly down-regulated the expression of cyclin D1 in a time- and concentration-dependent manners, and the phosphorylation of the downstream effector Rb was also inhibited. The expression of cyclin A and Cdk2 was down-regulated in the latter 12 h. However, DHC only slightly affected Cdk4 at any of the examined time points.

### Down-regulation of cyclin D1 by DHC causes growth inhibition via the Akt/GSK-3β pathway

**Figure 5:**
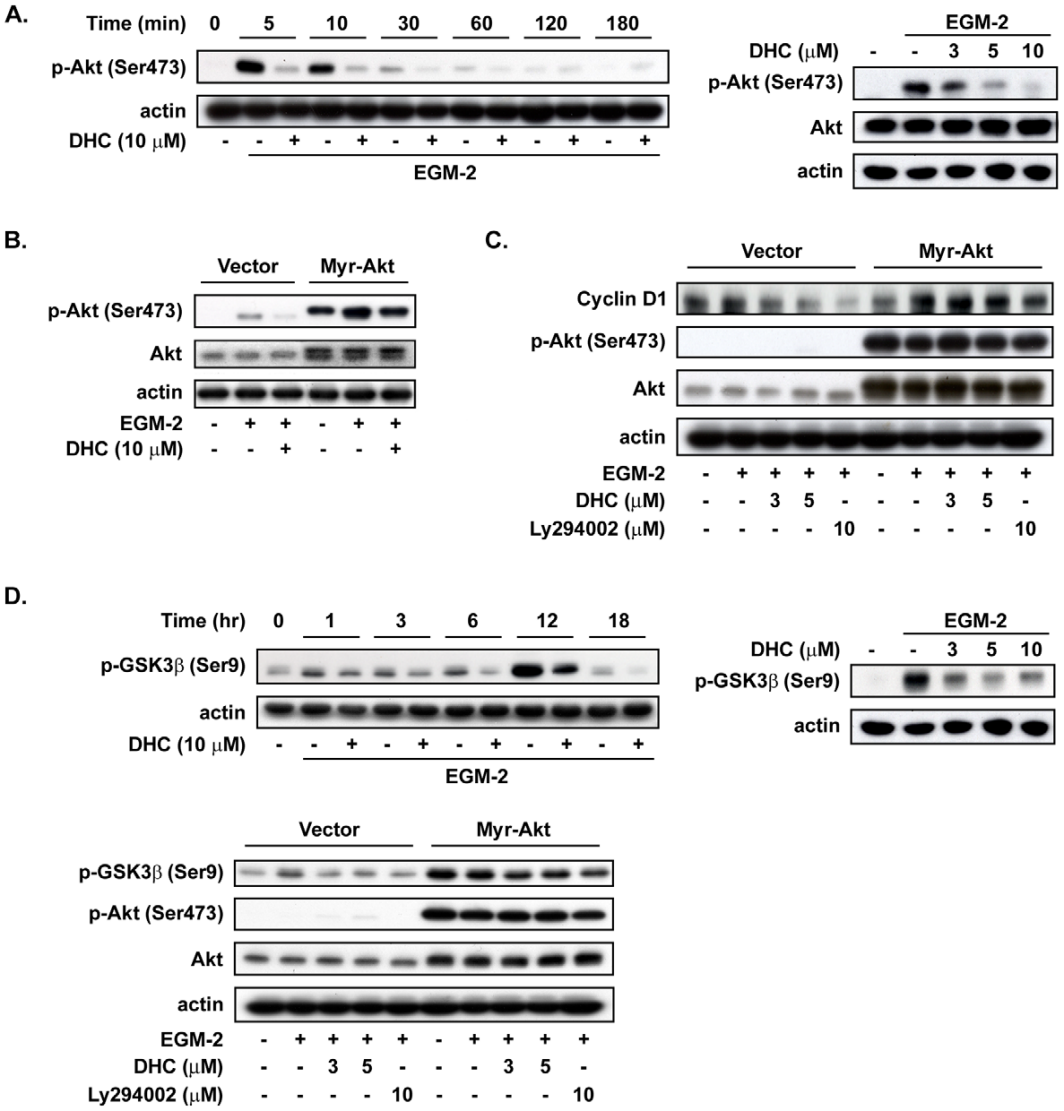
Cyclin D1 is a critical target of DHC-mediated cell cycle arrest through Akt/GSK-3β pathway. A, Western blot analysis of Akt phosphorylation inhibited by DHC with the indicated times and concentrations. B, HUVECs were transfected with either vector or myr-Akt, starved for 24 hr, and then pretreated with DHC for 1 hr followed by incubation in EGM-2 medium for 10 min. Cells were harvested and analyzed protein expression by western blot. C and D, western blot analysis of cyclin D1 expression and GSK-3β phosphorylation in HUVECs transfected with the indicated plasmids and then treated with DHC (3 and 5 µM) and Ly294002 (10 µM) for 6 hr. Data represent from three independent experiments.

Previous studies have shown that the serine/threonine kinase Akt signals in response to a variety of growth factors, including platelet-derived growth factor (PlGF), bFGF [24], [25], and VEGF [26]. To further delineate the mechanism that underlies the anti-angiogenic effect of DHC, the effect of DHC on Akt activation was examined by immunoblot analysis. Treatment with DHC dramatically suppressed EGM-2–induced Akt phosphorylation in the time- and concentration-dependent manners (Figure 5A). To verify that Akt was responsible for the proliferative response and the regulation of the expression of the cell cycle protein cyclin D1, we transiently transfected HUVECs with the constitutively active isoform of Akt and then determined its effect on DHC-induced growth inhibition. Transfection efficiency was confirmed, as shown in Figure 5B. LY294002- (an Akt inhibitor, used as a positive control) and DHC-treated HUVECs over-expressing Akt exhibited a highly significant increase in the expression of cyclin D1 relative to the vector-treated group (Figure 5C).

**Figure 6:**
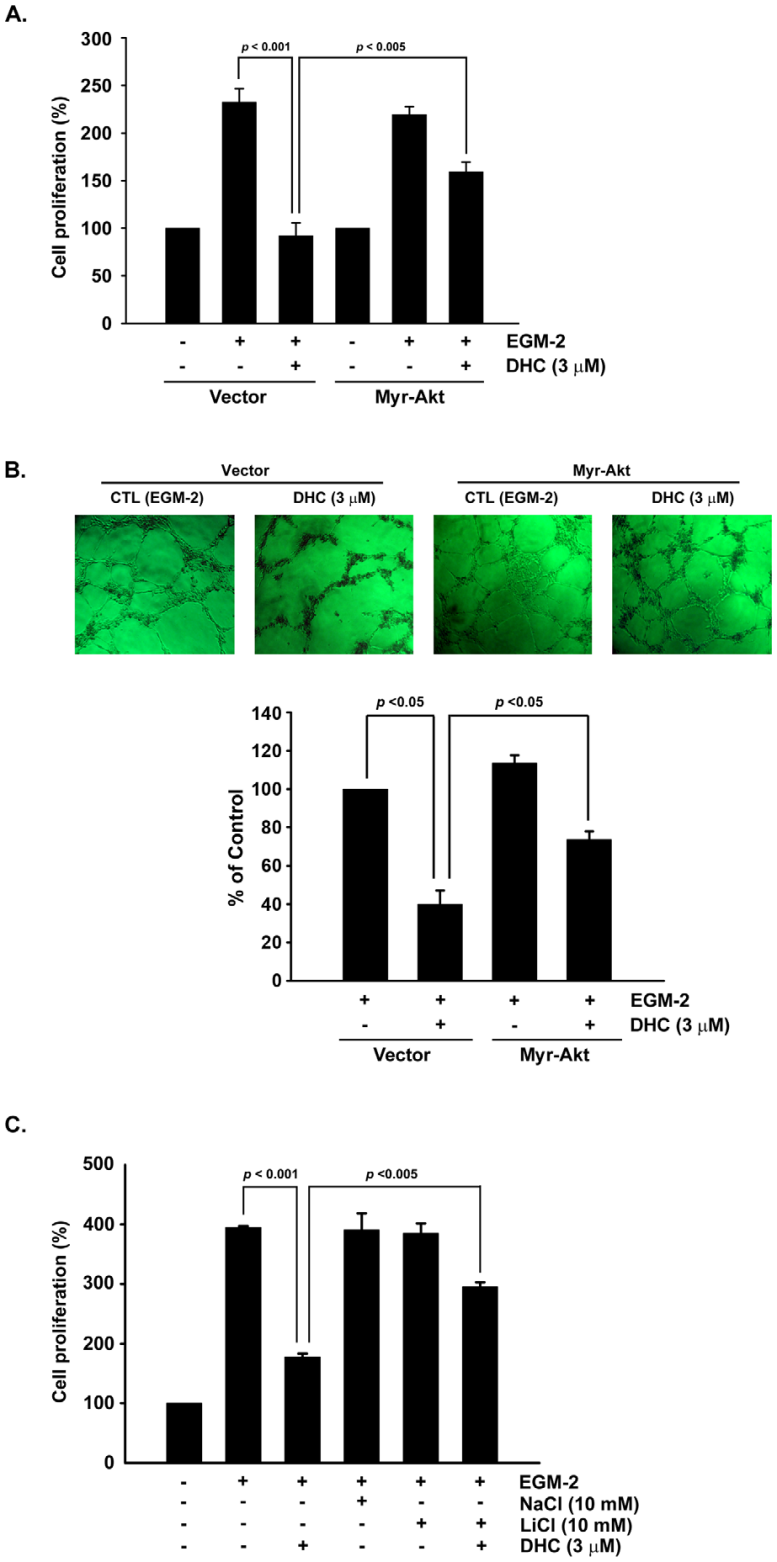
DHC inhibited cell proliferation and tube formation through Akt/GSK-3β pathway A, crystal violet assay. Treatment of vector group with DHC (3 µM) in EGM-2 medium significantly decreased cell proliferation numbers compared with untreated vector group. Akt overexpression significantly increased cell proliferation in DHC-treated group. B, tube formation assay. Treatment of vector group with DHC (3 µM) abrogated tube formation compared with vector control group. Akt overexpression partial reversed the inhibitory effect. C, crystal violet assay. HUVECs were treated with DHC (3 µM) and/or LiCl (10 mM). NaCl (10 mM) was as an osmolality control. Data represent from three independent experiments.

Akt signaling negatively regulates GSK-3β activity by phosphorylating Ser-9, and GSK-3β was shown to regulate the cell cycles through cyclin D1 proteasome-mediated degradation [8]. This study investigated whether the inhibition of Akt phosphorylation by DHC led to a decrease in GSK-3β phosphorylation levels and consequently contributed to growth inhibition. As shown in Figure 5D, treatment with DHC inhibited GSK-3β phosphorylation in the concentration- and time-dependent manners. However, DHC-treated cells combined with the over-expression of Akt increased the phosphorylation of GSK-3β compared with the vector group. As determined using the crystal violet assay, the growth inhibition induced by DHC was found to be reversed by myristoylated (myr)-Akt treatment (Figure 6A). Over-expression of Akt in HUVECs also partial reversed the abrogation of tube formation induced by DHC (Figure 6B). To verify whether GSK-3βis involved as a downstream effecter of Akt in the anti-angiogenic process of DHC, we treated HUVECs with LiCl, a GSK-3β activity inhibitor. Co-treatment of the LiCl with DHC significantly reversed the growth inhibition by approximately 50% of the DHC-treated cells (Figure 6C). Taken together, these results indicated that inhibition of the Akt/GSK-3β/cyclin D1 pathway plays an important role in DHC-induced growth inhibition and abrogation of tube formation in HUVECs.

### DHC suppresses expression of cyclin D1 in an mTOR-dependent manner

**Figure 7:**
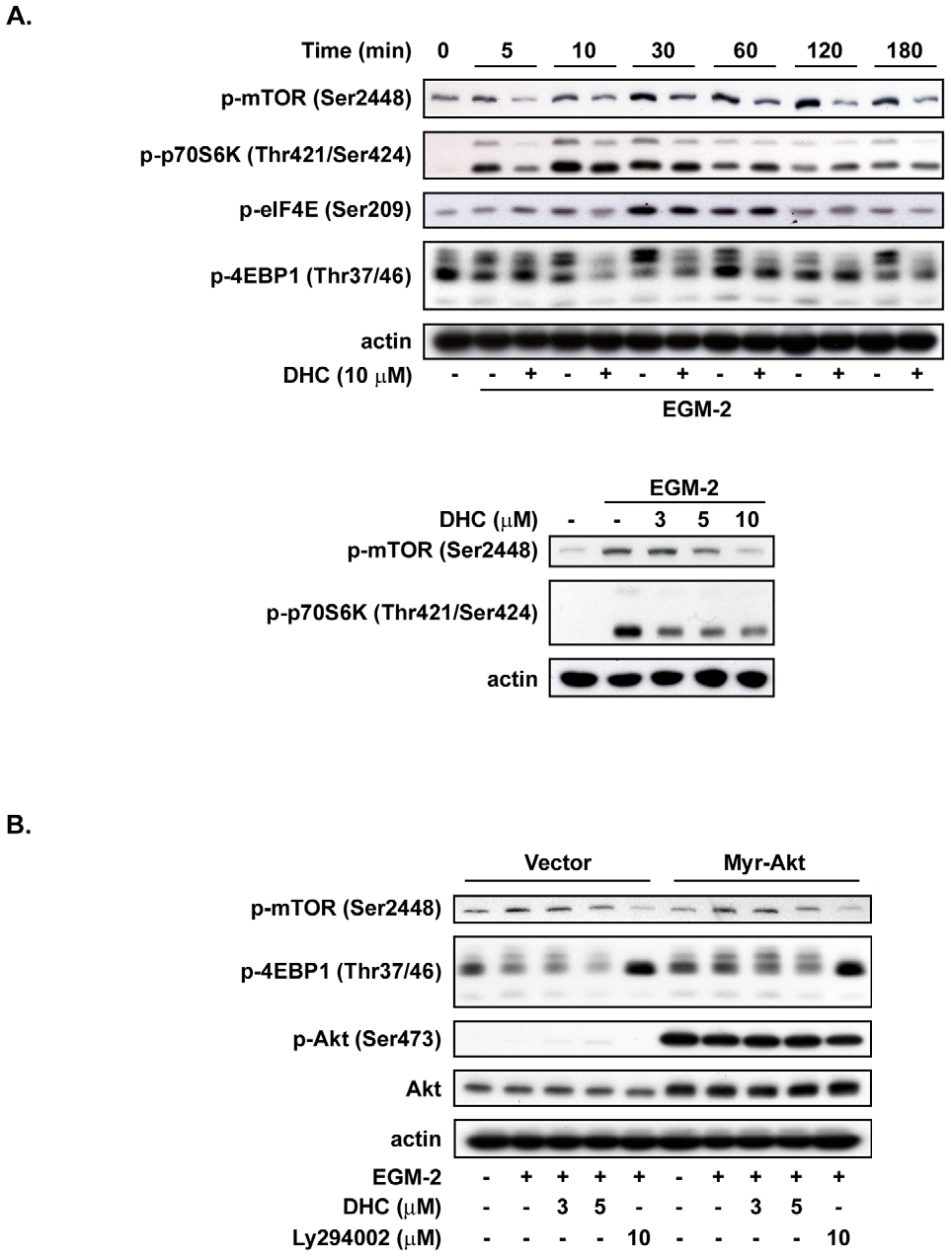
Effect of DHC on mTOR signaling downstream pathway. A, Western blot analysis of phosphorylation of mTOR, p70S6K, eIF4E and 4EBP in HUVECs treated with DHC for the indicated times and concentrations. B, after transfected with the indicated plasmids, HUVECs were starved for 24 hr and then pretreated with DHC followed by 10 min of EGM-2 incubation. Phosphorylation of mTOR and 4EBP were analyzed by western blot. Data represent from three independent experiments.

Akt signaling is required for growth factor-induced activation of mammalian target of rapamycin (mTOR)-C1, and the activity of mTORC1 is impaired in Akt-deficient cells [27], [28]. The activation of mTORC1 also leads to the phosphorylation of 4-emopamil-binding protein (EBP)-1 and, therefore, activates eIF4E to up-regulate the translational levels of cyclin D1 [29]. To evaluate the contribution of mTORC1 in the DHC-induced downregulation of cyclin D1 in HUVECs, the downstream signaling effectors of mTORC1 were examined. We found that DHC effectively suppressed EGM-2-induced phosphorylation of the mTOR signaling cascade, including mTOR, p70S6K, 4EBP, and eIF4E, in a time-dependent manner (Figure 7A). DHC also inhibited the phosphorylation of mTOR downstream molecules in a concentration-dependent manner (Figure 7A). In HUVECs overexpressing Akt, the inhibitory effects of DHC on the phosphorylation of mTOR downstream signaling molecules had significantly reversed protein expression relative to the vector group (Figure 7B). Using the crystal violet assay, the co-treatment of rapamycin and DHC increased the growth inhibition effect compared with DHC alone (Figure S3). These results indicated that the Akt/mTOR signaling pathway could be involved in the growth arrest induced by DHC inhibition of cyclin D1.

## Discussion

Tumor progression and metastatic shedding are dependent on angiogenesis [30]. Thus, in recent years, the development of anti-angiogenic agents has become attractive. Additionally, natural products have been reported to have a cytotoxic effect on the normal cell cycle. However, thus far, out of 22 anti-angiogenic agents, there are only 11 natural products or natural modified compounds have been under clinical trials [31]. This study found that DHC, a component of traditional Chinese medicine, has anti-angiogenic effects *in vivo* and *in vitro*. First, this study utilized the matrigel plug assay, which provided strong evidence that DHC inhibited angiogenesis *in vivo* in a dose-dependent manner. Previous studies have revealed that DHC suppresses the growth of various human tumors in xenograft mouse models [21], [22], [23]. Therefore, the inhibition of angiogenesis by DHC seems to be one of the main mechanisms that may hinder tumor progression. A series of *in vitro* assays established that the inhibition of EGM-2-induced tube formation and endothelial cell proliferation were consistent with this study's *in vivo* result. In contrast, DHC had no significant inhibitory effect on cell migration in the transwell assay. Using the western blot, activation of p38 and ERK1/2 signaling has been observed when HUVECs were treated with DHC (figure S1B). We employed another system, a scratch wound healing assay to double confirm the effect of DHC on HUVECs migration. However, only combined treatment with p38 and DHC significantly inhibited HUVECs migration (figure S1A). The result indicated that even with inhibition of PI3K/Akt pathways, p38 activation contributed to the signaling of HUVECs migration while Akt signaling is inhibited. Taken together, we suggested that DHC blocked blood vessel formation by the selective inhibition of endothelial cell growth and capillary-like structure formation.

Cell proliferation is a critical event in the process of angiogenesis. This study found that DHC delayed the transition of HUVECs from the G0/G1 phase to the S phase. These results are consistent with those of a previous study, which found that DHC treatment induced growth inhibition in breast cancer [23]. Cyclin D1, known as an essential mitogenic signal sensor and cell cycle regulator, binds to CDK4 and forces cells to enter the proliferative stage of cell cycle from the G0 phase. Cyclin D1 reportedly plays an important role in endothelial cells. More recent studies have demonstrated that overexpression of cyclin D1 is linked to the development of various cancers [12]. Therefore, strategies to down-regulate cyclin D1 expression were reported to inhibit tumor angiogenesis *in vitro* and *in vivo* [13], [32]. Our results indicated that DHC significantly inhibited cyclin D1 expression within 3 h of DHC treatment in a concentration- and time-dependent manner. Other cell cycle regulators appeared to be down-regulated after 12 h of DHC treatment. This study concluded that DHC-induced growth arrest of HUVECs via the down-regulation of cyclin D1, and it might play an important role in the suppression of angiogenesis.

Evidence implies that Akt signaling is essential for normal cellular G1/S phase transition and cell proliferation in endothelial cells [33], [34], [35], [36]. Data from preclinical studies suggest that inhibitors of the PI3K/Akt signaling pathway have been reported to be potent anti-angiogenic agents and contribute to the inhibition of tumor growth [37], [38], [39], [40], [41]. Upon growth factor stimulation, Akt is activated immediately in endothelial cells. The phosphorylation of Akt at the T308 residue was mediated by PDK1, whereas phosphorylation at the S473 residue was attributed to mTORC2 [42]. The two phosphorylation sites are equally important, and lead to full activation of the Akt protein kinase. The major effectors downstream of Akt are mTORC1 and GSK-3β. The active mTORC1 induces cyclin D1 expression to promote cell growth by direct phosphorylation of the substrates, S6K1 and 4EBP1, both of which are translational regulators [29], [43]. Alternatively, Akt phosphorylates and inhibits GSK-3β activity to prevent cyclin D1 degradation [7], [8]. Therefore, inhibition of the Akt signaling pathway resulted in the down-regulation of cyclin D1 via a GSK-3β-degradation and mTORC1-dependent translation pathway. Most importantly, this study has identified, for the first time, the effect of DHC on the Akt/GSK-3β/cyclin D1 pathway. Additionally, DHC inhibited Akt phosphorylation at Ser473 in HUVECs, in a concentration- and time- dependent manner, suggesting that DHC is a possible competitor of mTORC2. As mentioned above, the activation of Akt is induced by various growth factors in a PI3K-dependent manner. In this study, we found that treatment with myr-Akt greatly increased the amount of Ser473 Akt present in HUVECs. Our results are similar to those for treatment with the PI3K inhibitor LY294002. We also performed the Akt kinase activity assay. However, DHC did not inhibit the ability of Akt to phosphorylate its substrate (figure S2). Therefore, DHC is a possible potent PI3K kinase or PDK1 kinase inhibitor that could display anti-angiogenic effects *in vitro* and *in vivo*.

Previous studies have shown that mTORC1 activation is not only under the regulation of Akt signaling but is also sensitive to growth factors, hypoxia, low energy, and amino acids, and it integrates these inputs to regulate cell responses [44]. However, the results showed that myr-Akt can reverse the inhibitory effects of DHC on the mTOR pathway. This study suggests that the Akt/mTOR translational pathway is another important target responsible for DHC-induced cyclin D1 down-regulation. Previous studies have reported other targets of DHC in cancer cells including NF-κB, STAT3, and induction of ER stress [20], [21], [23]. In addition, activation of NF-κB has been known to regulate the expression of genes encoding angiogenic factors. Therefore, the contribution of these targets to the anti-angiogenic effect of DHC cannot be excluded. However, overexpression of myr-Akt significantly rescued the growth inhibition induced by DHC, suggesting that DHC-induced inhibition of Akt activation is responsible for the anti-proliferative effect observed in HUVECs. Thus, the mechanisms of DHC mentioned above may probably synergistically exhibit anti-angiogenic activity *in vitro* and *in vivo*.

In conclusion, this study discovered a novel mechanism by which DHC impairs angiogenesis induced by growth factors. DHC-induced cell cycle in HUVECs was arrested at the G0/G1 phase, followed by subsequent inhibition of angiogenesis *in vitro* and *in vivo* by targeting the Akt/GSK-3β and mTOR pathway. Thus, the results generated by this study suggest that DHC is a promising traditional Chinese medicine component for therapeutic intervention against angiogenesis-related diseases.

## Materials and Methods

### Reagents

Dehydrocostuslactone (DHC) was purchased from Wako Pure Chemical Industries, Ltd. (Osaka, Japan). Propidium iodide was obtained from Sigma. Medium 199, fetal bovine serum (FBS), penicillin, streptomycin and the other tissue culture reagents were obtained from Gibco BRL Life Technologies (Grand Island, NY). Endothelial cell basal medium (EBM) and endothelial growth factors (EGM-2) were purchased from Clonetics (BioWhittaker, Walkersville, MD). The antibody against cyclin D was purchased from Calbiochem (San Diego, CA). Antibodies against CDK2, CDK4, cyclin A were purchased from Santa Cruz Biotechnology. Antibodies against p-mTOR (Ser2448), p-p70S6K (Thr421/Ser424), p-eIF4E (Ser209), p-4EBP1 (Thr37/46), p-GSK-3β (Ser9) and Akt were purchased from Cell Signaling Technology. The antibody against p-Akt (Ser473) was purchased from Epitomics (San Francisco, CA, USA).

### Cell culture

Human umbilical vein endothelial cells (HUVEC) (ScienCell Research Laboratories, San Diego, CA) were grown to confluence on 1% gelatin, and maintained in endothelial cell medium (ECM) (ScienCell Research Laboratory) supplemented with 5% FBS, 1∶5 Penicillin/Streptomycin (ScienCell Research Laboratory) and 1∶5 endothelial cell growth supplements (ECGs) (ECGs; Upstate Biotechnology Inc., Lake Placid, NY). Before treatment of dehydrocostuslactone in EGM-2 medium, HUVECs were starved for 24 hours in EBM-2 medium. All of the following experiments were performed in this medium.

### *In vivo* matrigel plug assay

All animal experiments followed ethical standards, and protocols has been reviewed and approved by Animal Use and Management Committee of National Health Research Institutes (NHRI-IACUC-099003-A). *In vivo* angiogenesis assay was determined as blood vessel growth in the exogenous matrigel plug injected in C57BL/6 mice (6 weeks old). Briefly, matrigel (BD Bioscience) was mixed with heparin (10 units/ml), VEGF (40 ng/ml), IGF-1 (40 ng/ml), EGF (40 ng/ml), bFGF (40 ng/ml) and with or without DHC and the resulting mixture was injected subcutaneously into the mice abdomens. After seven days, the animals were sacrificed and the matrigels were carefully dissected and photographed. To quantify the blood vessel formation, hemoglobin content was analyzed by Drabkin's reagent kit (Sigma).

### Crystal violet assay

The HUVECs were seeded into 96-well culture plates (5.0×10^3^ cells per well) in triplicate. Twenty-four hours later, the culture medium was removed and replaced with fresh EGM-2 medium containing DHC at various concentrations. After 72 hours of incubation at 37°C, the cells were stained with 0.1% crystal violet/20% methanol for 10 minutes. Then the dye was eluted by 0.1 M sodium citrate/75% ethanol, and absorbance is measured at 540 nm with an ELISA reader.

### DNA synthesis assay

Cell growth was measured by 5-bromo-2-deoxyuridine (BrdU) incorporation into newly synthesized DNA of proliferating HUVECs with the colorimetric ELISA kit (Amersham Biosciences, Piscataway, NJ). Briefly, cells were seeded at 5.0×10^3^ cells per well into 96-well culture plates in triplicate and starved with EBM-2 medium overnight. Then the cells were treated with or without DHC and EGM-2 medium for 48 hours. To label the synthesized DNA, 10 µM BrdU was added to cultures for the last 18 hours before staining. After incubation, the cells were fixed and detected with anti-BrdU mAb according to the manufacturer's instructions. The absorbance was measured at 450 nm.

### Endothelial cell migration assay

The assessment of HUVEC cells migration was performed by using Transwell (Corning) with 8 µM pore sized filters. First, HUVECs (75000 cells/100 µl) were pretreated with DHC for 30 minutes and then plated in the upper chamber of the Transwell. To allow for cell migration, EGM-2 medium was added to the lower chamber as chemoattractant. After 6 hours of incubation at 37°C, the nonmigrated cells in the upper chamber were wiped away with cotton swabs and cells that migrated to the lower surface of the filters were fixed and stained with crystal violet, and photographed with a microscope. The filters were cut from the chamber and then eluted with 0.1 M sodium citrate solution to quantify the migrated cells. The absorbance was measured at 540 nm.

### Capillary tube formation assay

The assay was performed by utilizing Matrigel (Growth factor reduced, BD Biosciences) added to a 96 well plate and the gel allowed to solidify at 37°C for 1 hour. Subsequently, HUVECs were harvested after trypsin treatment, were then resuspended in EGM-2 medium in the presence or absence of DHC at various concentrations, seeded onto the layer of Matrigel at a cell number of 1.75×10^4^/per well. After 6 hours of incubation, tubular network structures were visualized and photographed under a phase contrast microscope. The relative lengths of tubes were quantified by Image analysis software (Image-Pro® Plus).

### Flow cytometric analysis

HUVECs grown to 70% confluence were starved with EBM-2 medium for 24 hours and then replaced with EGM-2 medium with or without DHC for 18 hours. After trypsinized and fixed in ice-cold 75% methanol for a minimum of one hour at −20°C, cells were washed with PBS and resuspended in 0.2 ml DNA extraction buffer (0.2 M Na_2_HPO_4_, 0.1 M citric acid; pH 7.8) for 30 minutes. Then the cells were stained with propidium iodide solution (PI; 100 µg/ml RNase, 80 µg/ml propidium iodide, 0.1% Triton X-100) in PBS. Cell cycle distribution was determined by FACScan flow cytometry, and data analysis was performed with CellQuest software (BD Biosciences).

### Western blotting

HUVECs were harvested after treatment of DHC in EGM-2 medium at an indicated time, and then total cell lysate were prepared in a modified RIPA buffer (150 mM NaCl, 1 mM EDTA, 1% Nonidet p-40, 0.5% sodium deoxycholate, 0.1% SDS, 20 mM Tris, pH 8.0) with protease inhibitors (1 µg/ml aprotinin, 1 µg/ml leupeptin, 0.5 M NaF, 0.5 M Na_3_VO_4_, 1 mM phenylmethylsulfonyl fluoride). The cell extract proteins were separated by 8%–12% polyacrylamide gel electrophoresis followed by electroblotting onto polyvinylidene difluoride membranes. Membranes were subsequently blocked with 5% nonfat milk, washed with PBS, incubated with antibodies and detected utilizing an enhanced chemiluminescence (ECL) detection system.

### Statistical analysis

The significance of differences *in vivo* data were analyzed by the Mann-Whitney U test; others represent the mean and SEM of at least three independent experiments. Statistical analysis was performed by the *t*-test, and *P* values less than 0.05 (* *P*<0.05, ** *P*<0.01, *** *P*<0.001) were considered significant.

## Supporting Information

Figure S1 **Effect of DHC on HUVECs migration.** A, HUVECs migration after DHC treatment was accessed by wound-healing assay. Upon reaching 95% confluence, the HUVEC monolayer was scratched and cell debris was removed. Cells were cultured with EGM-2 medium and preteated with PD98059 (10 µM) or SB203580 (10 µM) for 30 min, and then cells were treated with DHC (3 or 5 µM). After incubation for 16 h, cells were stained with crystal violet and photographed. B, Western blot analysis of the protein expression of p-p38, p-ERK1/2, CHOP in DHC-treated HUVECs with the indicated times and concentrations. Data represent from three independent experiments.
(PDF)

Figure S2 **DHC did not inhibit Akt kinase activity.** Akt Kinase activity kit was purchased from Enzo Life Sciences. Data represent from three independent experiments.
(PDF)

Figure S3 **Rapamycin increased the anti-proliferative effect induced by DHC.** Crystal violet assay. HUVECs were treated with DHC (3 µM) and/or rapamycin (3 µM). Inhibition of mTOR activity increased the anti-proliferative effect of DHC. Data represent from three independent experiments.
(PDF)

Figure S4 **Effect of DHC on early and late stage of HUVECs apoptosis was detected by flow cytometry with Annexin-V-FITC/PI dual staining.** A representative histogram of flow cytometric analysis using double staining with annexin-V (FITC-A) and PI (PE-A). HUVECs were treated with DHC (5 µM) in EBM-2 basal medium for 4 hr or 24 hr. The lower right quadrants represent the cells in the early stage of apoptosis. The upper right plus left quadrants contain the cells in the late stage of apoptosis and necrosis. Data represent from three independent experiments.
(PDF)
