# Supplementary material for: Dehydrocostuslactone Suppresses Angiogenesis In Vitro and In Vivo through Inhibition of Akt/GSK-3β and mTOR Signaling Pathways
Source: PLoS One. 2012 Feb 16;7(2):e31195. doi: 10.1371/journal.pone.0031195 (PMC3281050; doi:10.1371/journal.pone.0031195)
Supplement: Figure S1 — Effect of DHC on HUVECs migration. A, HUVECs migration after DHC treatment was accessed by wound-healing assay. Upon reaching 95% confluence, the HUVEC monolayer was scratched and cell debris was removed. Cells were cultured with EGM-2 medium and preteated with PD98059 (10 µM) or SB203580 (10 µM) for 30 min, and then cells were treated with DHC (3 or 5 µM). After incubation for 16 h, cells were stained with crystal violet and photographed. B, Western blot analysis of the protein expression of p-p38, p-ERK1/2, CHOP in DHC-treated HUVECs with the indicated times and concentrations. Data represent from three independent experiments. (PDF) [file pone.0031195.s001.pdf]

Chih-Ya Wang et al.
Supplemental Figure S1

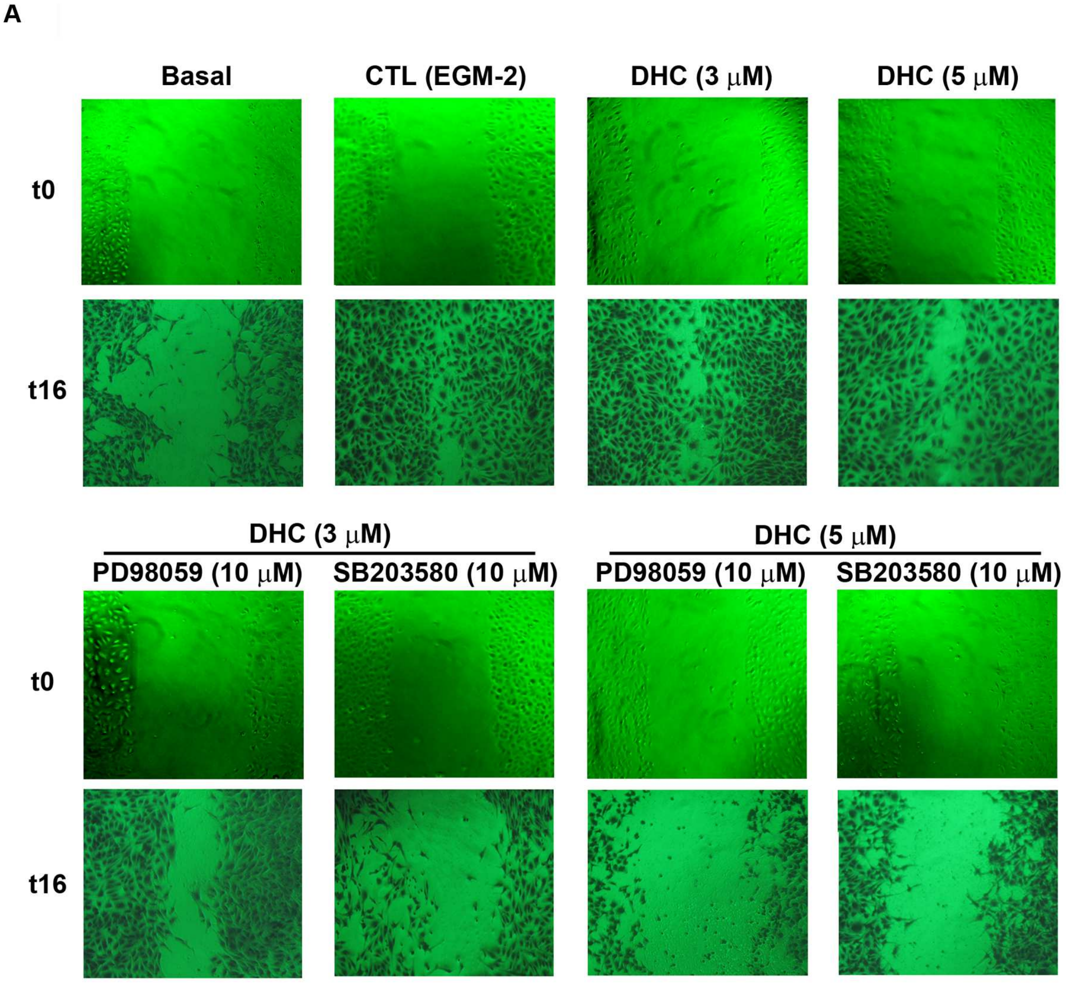

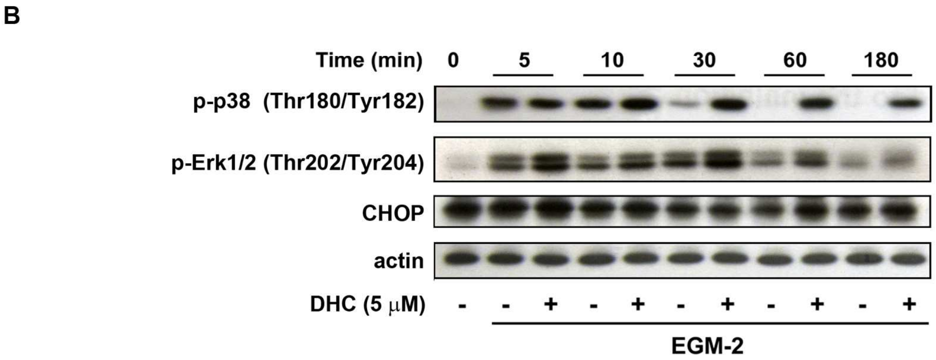

Supplemental Figure S1. Effect of DHC on HUVECs migration. A, HUVECs migration after DHC treatment was accessed by wound-healing assay. Upon reaching 95% confluence, the HUVEC monolayer was scratched and cell debris was removed. Cells were cultured with EGM-2 medium and preteated with PD98059 (10 μM) or

SB203580 (10 μM) for 30 min, and then cells were treated with DHC (3 or 5 μM). After incubation for 16 h, cells were stained with crystal violet and photographed. B, Western blot analysis of the protein expression of p-p38, p-ERK1/2, CHOP in DHC-treated HUVECs with the indicated times and concentrations. Data represent from three independent experiments.
