# Supplementary material for: Dehydrocostuslactone Suppresses Angiogenesis In Vitro and In Vivo through Inhibition of Akt/GSK-3β and mTOR Signaling Pathways
Source: PLoS One. 2012 Feb 16;7(2):e31195. doi: 10.1371/journal.pone.0031195 (PMC3281050; doi:10.1371/journal.pone.0031195)
Supplement: Figure S2 — DHC did not inhibit Akt kinase activity. Akt Kinase activity kit was purchased from Enzo Life Sciences. Data represent from three independent experiments. (PDF) [file pone.0031195.s002.pdf]

Chih-Ya Wang et al.
Supplemental Figure S2

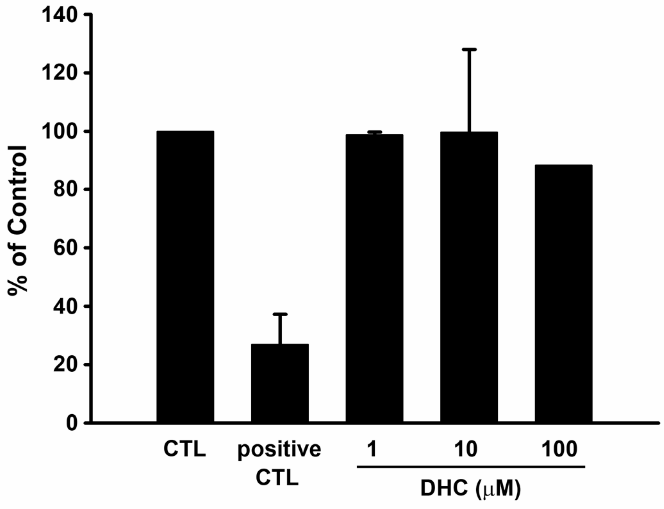

Supplemental Figure S2. DHC did not inhibit Akt kinase activity. Akt Kinase activity kit was purchased from Enzo Life Sciences. Data represent from three independent experiments.
