# Supplementary material for: Dehydrocostuslactone Suppresses Angiogenesis In Vitro and In Vivo through Inhibition of Akt/GSK-3β and mTOR Signaling Pathways
Source: PLoS One. 2012 Feb 16;7(2):e31195. doi: 10.1371/journal.pone.0031195 (PMC3281050; doi:10.1371/journal.pone.0031195)
Supplement: Figure S3 — Rapamycin increased the anti-proliferative effect induced by DHC. Crystal violet assay. HUVECs were treated with DHC (3 µM) and/or rapamycin (3 µM). Inhibition of mTOR activity increased the anti-proliferative effect of DHC. Data represent from three independent experiments. (PDF) [file pone.0031195.s003.pdf]

Chih-Ya Wang et al.
Supplemental Figure S3

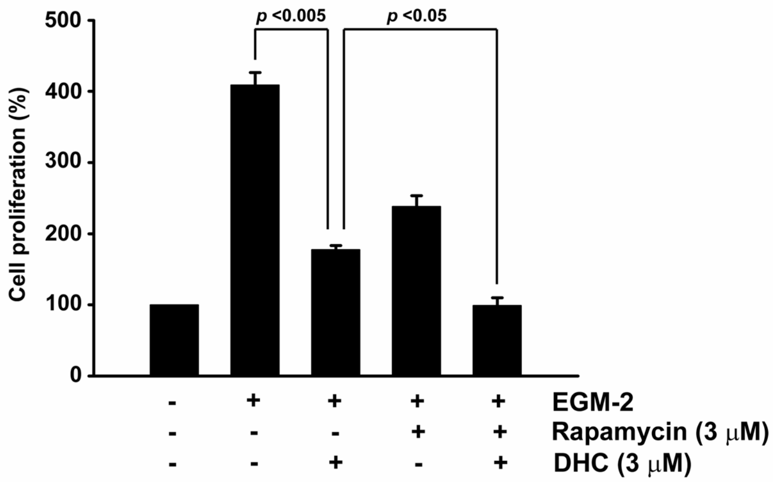

Supplemental Figure S3. Rapamycin increased the anti-proliferative effect induced by DHC. Crystal violet assay. HUVECs were treated with DHC (3 μM) and/or rapamycin (3 μM). Inhibition of mTOR activity increased the anti-proliferative effect of DHC. Data represent from three independent experiments.
