# Supplementary material for: Dehydrocostuslactone Suppresses Angiogenesis In Vitro and In Vivo through Inhibition of Akt/GSK-3β and mTOR Signaling Pathways
Source: PLoS One. 2012 Feb 16;7(2):e31195. doi: 10.1371/journal.pone.0031195 (PMC3281050; doi:10.1371/journal.pone.0031195)
Supplement: Figure S4 — Effect of DHC on early and late stage of HUVECs apoptosis was detected by flow cytometry with Annexin-V-FITC/PI dual staining. A representative histogram of flow cytometric analysis using double staining with annexin-V (FITC-A) and PI (PE-A). HUVECs were treated with DHC (5 µM) in EBM-2 basal medium for 4 hr or 24 hr. The lower right quadrants represent the cells in the early stage of apoptosis. The upper right plus left quadrants contain the cells in the late stage of apoptosis and necrosis. Data represent from three independent experiments. (PDF) [file pone.0031195.s004.pdf]

Chih-Ya Wang et al.
Supplemental Figure S4

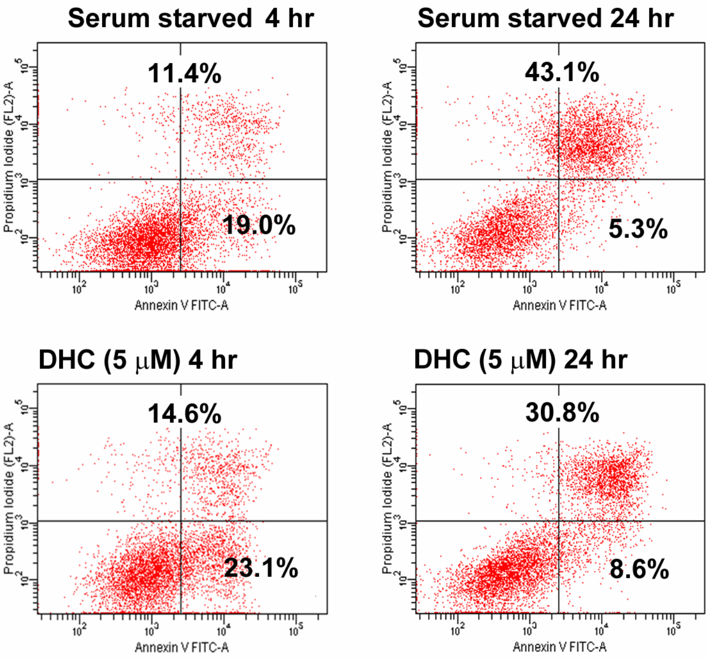

Supplemental Figure S4. Effect of DHC on early and late stage of HUVECs apoptosis was detected by flow cytometry with Annexin-V-FITC/PI dual staining. A representative histogram of flow cytometric analysis using double staining with annexin-V (FITC-A) and PI (PE-A). HUVECs were treated with DHC (5 μM) in EBM-2 basal medium for 4hr or 24hr. The lower right quadrants represent the cells in the early stage of apoptosis. The upper right plus left quadrants contain the cells in the late stage of apoptosis and necrosis. Data represent from three independent experiments.
